# *Clock* Gene Modulates Roles of *OXTR* and *AVPR1b* Genes in Prosociality

**DOI:** 10.1371/journal.pone.0109086

**Published:** 2014-10-13

**Authors:** Haipeng Ci, Nan Wu, Yanjie Su

**Affiliations:** 1 Department of Psychology, Peking University, Beijing, P.R. China; 2 Teachers’ College of Beijing Union University, Beijing, P.R. China; Karlsruhe Institute of Technology, Germany

## Abstract

**Background:**

The arginine vasopressin receptor (*AVPR*) and oxytocin receptor (*OXTR*) genes have been demonstrated to contribute to prosocial behavior. Recent research has focused on the manner by which these simple receptor genes influence prosociality, particularly with regard to the AVP system, which is modulated by the *clock* gene. The *clock* gene is responsible for regulating the human biological clock, affecting sleep, emotion and behavior. The current study examined in detail whether the influences of the *OXTR* and *AVPR1b* genes on prosociality are dependent on the *clock* gene.

**Methodology/Principal Findings:**

This study assessed interactions between the *clock* gene (rs1801260, rs6832769) and the *OXTR* (rs1042778, rs237887) and *AVPR1b* (rs28373064) genes in association with individual differences in prosociality in healthy male Chinese subjects (*n* = 436). The Prosocial Tendencies Measure (PTM-R) was used to assess prosociality. Participants carrying both the GG/GA variant of *AVPR1b* rs28373064 and the AA variant of *clock* rs6832769 showed the highest scores on the Emotional PTM. Carriers of both the T allele of *OXTR* rs1042778 and the C allele of *clock* rs1801260 showed the lowest total PTM scores compared with the other groups.

**Conclusions:**

The observed interaction effects provide converging evidence that the *clock* gene and OXT/AVP systems are intertwined and contribute to human prosociality.

## Introduction

Increasing evidence suggests that circadian rhythms are important regulatory processes. Most organisms form and maintain daily patterns of behavior to adapt to 24-h cycles of light and temperature in their environment [1], [2]. The molecular mechanisms of the circadian cycle involve at least 9 core circadian genes that control transcriptional and translational feedback loops [3], encoding activator and repressor proteins. Among these circadian genes, the circadian locomotor output cycles kaput (*clock*) gene is transcribed to produce the CLOCK protein, which is an essential element of the circadian pacemaker that plays a vital role in regulating human biological timing [4]. The circadian regulatory loop consists of positive elements CLOCK and BMAL1 which bind as a heterodimer to an enhancer element termed the E-box. Notably, to better anticipate and adapt to daily environmental changes, CLOCK:BMAL1 heterodimer expression levels rise to activate the transcription of clock-controlled genes during the day, whereas these levels decrease at night, thereby reducing the transcription of the clock-controlled genes [1], [3]. *Clock* mRNA and protein are constitutively expressed in the suprachiasmatic nucleus (SCN), which is the principle circadian oscillator [5]. Furthermore, the *clock* gene and SCN-mediated circadian clock output affect sleep and emotion [6]. For example, rs1801260 (3111T/C), which is a SNP located in the 3′-flanking region of the *clock* gene, has been shown to contribute to bipolar disorders and sleep disorders, whereas CC carriers are more likely to suffer from both bipolar and sleep disorders [7], [8]. Additionally, previous studies have reported that C allele carriers of rs1801260 display more emotional apathy than the TT carriers [9]. Furthermore, the C (minor) allele is associated with evening preference among Caucasian and Asian populations [10], [11], with evening hours being favored for novelty-seeking and impulsive activities, which leads to reduced affiliating emotions and prosocial behavior [12]. Moreover, a genome-wide association study by Terracciano et al. [13] indicated that the *clock* SNPs rs1801260 (3111T/C) and rs6832769 show the strongest associations with prosocial behavior as recognized by agreeableness, which is one of the five broad dimensions of human personality. Because the heritability of prosocial behavior has been emphasized both in twin-designed studies and molecular genetic studies [14], [15] and considering the aforementioned evidence, we aimed to explore the relationship between *clock* gene and prosocial behavior, although little direct evidence exists suggesting that they are linked.

Current knowledge indicates that the core clock mechanism involves E-box-regulated transcription. The transcriptional activators CLOCK and BMAL1 bind as heterodimers to CACGTG E-box enhancers located in the promoters of the *per*, *cry*, and clock-controlled genes, to modulate the functions of the central clock in the brain [1]. Indeed, the expression levels of many genes are regulated by CLOCK:BMAL1 heterodimers acting through E-box elements [16]. The product of one such clock-controlled gene, arginine vasopressin (AVP), contributes to extracellular signaling and dopamine metabolism, controlling behavioral and neuroendocrine cycles [17]. Among the human vasopressin receptors, the arginine vasopressin V1b receptor (AVPR1b) is important in regulating the responsiveness of pituitary corticotrophins to vasopressin. AVPR1b is expressed primarily in the pituitary and discrete areas of the brain, including the SCN, which is a fundamental area where clock-controlled genes are also expressed [18]. Evidence suggests that the *AVPR1b* gene is closely related to anxiety and depression [19]. For example, changes in pituitary AVPR1b level contribute to corticotrophin responsiveness under chronic stress, and the up-regulation of the AVPR1b has been suggested in individuals with depression, which could contribute to the shift in the hypothalamic drive from corticotrophin-releasing hormone to AVP [20]. Furthermore, the genotypic variation *AVPR1b* rs28373064 may disturb the sleep-wake cycle and other circadian rhythms, which may cause problems with vasopressin, thus affecting mood [21]. Because prosociality may serve as a coping strategy for reducing depression and stress [22], we hypothesized that variations in the *AVPR1b* gene are related to prosociality and that this relation might be modulated by the *clock* gene.

Oxytocin (OT) is another nonapeptide that shares a similar chemical structure with AVP. The two most important social hormones, AVP and OT, are both nonapeptides synthesized in the hypothalamus and released into the bloodstream via axon terminals in the posterior pituitary or neurohypophysis. Most regions that express *AVPR1b* mRNA also express oxytocin receptor (*OXTR*) mRNA [18]. Furthermore, OT and AVP are known to mediate affiliative behaviors in mammals [23]. Recently, OT has increasingly been established as a prosocial neuropeptide in humans due to its close relationship with personal trust, generosity and charitable giving [23]. The *OXTR* gene contributes to empathy and prosociality. For example, carriers homozygous for the G allele of *OXTR* rs237887 display more emotional empathy than those with the A allele [24], and GG carriers are also more prosocial than AA carriers [15]. A significant association was also observed with carriers of the G allele of the rs1042778 SNP, who showed higher levels of giving in the dictator game [15]. Feldman et al. [25] showed that the GG genotype of the SNP rs1042778 is associated with increased affiliative behaviors and generosity. In these aforementioned studies, the individuals with the GG genotypes were found to display increased prosociality; thus, it is termed as the “generous” genotype. In contrast, individuals with the A allele exhibited decreased prosociality, and the associated genotype is termed the “mean” genotype. Therefore the genotypes (e.g., AA genotype of *OXTR* rs1042778) associated with decreased prosociality include the “risk alleles” for prosociality [24], [25]. Ebstein, Israel, Chew, Zhong, and Knafo [26] have advocated studies exploring gene × gene interactions in prosocial behavior; thus, we hypothesized that the link between the *OXTR* gene and prosocial behavior may be modulated by the *clock* gene.

Therefore, to address the impact of gene interactions on prosociality and to clarify the association between the *clock* gene and prosociality, 2 SNPs of the *clock* gene (rs1801260 and rs6832769) and an additional 3 SNPs of the *OXTR* (rs1042778 and rs237887) and *AVPR1b* (rs28373064) genes were selected. The present study had two hypotheses; first, that the *clock* gene is closely related to prosociality; and second, that the influences of the *OXTR* and *AVPR1b* genes on prosociality are modulated by the *clock* gene.

## Materials and Methods

### Participants

In total, 436 healthy male college students were recruited, the mean age was 21.84 (*SD* = 1.44). The participants first provided buccal swabs for the genotyping of *OXTR* rs1042778 and rs237887, *AVPR1b* rs28373064, and *clock* rs1801260 and rs6832769. Subsequently, all participants completed a paper-and-pencil version of the Prosocial Tendencies Measure (PTM-R). All participants gave written informed consent prior to the study. Upon completion of all tests, a gift was given for their participation. The study was approved by the local ethics committees of Peking University.

### Prosocial Tendencies Measure

The PTM-R [27], [28] was used to assess six different prosocial behavioral tendencies that tend to vary according to situation (e.g., emergency situations) and motive (e.g., altruism). The 26-item version of the PTM-R was composed of 6 subscales: Public (4 items), Anonymous (5 items), Dire (3 items), Emotional (4 items), Compliant (5 items) and Altruism (4 items). The participants were asked to rate the extent to which the statements described themselves on a 5-point scale ranging from 1 (does not describe me at all) to 5 (describes me greatly). Previous research demonstrated that the Chinese version of the PTM-R has adequate internal reliability and validity [28]. The present study also showed adequate internal reliability for this test, with alpha levels ranging from 0.67–0.82.

### Genotyping

DNA was extracted from saliva collected into Oragene Saliva Kits (DNA Genotek Inc., Beijing, China) using the Agencourt DNAdvance Kit (TianGen Biotech Ltd., Beijing, China). Based on previous reports, the *clock* gene SNPs rs6832769 and rs1801260, *OXTR* gene SNPs rs1042778 and rs237887 and *AVPR1b* gene SNP rs28373064 were genotyped using polymerase chain reaction–restriction fragment length polymorphism (PCR-RFLP) analysis. The MassArray PCR primer and probe sets for the SNPs were obtained from Assays-On-Demand (www.sequence.com). The SNPs were genotyped using the MassArray genotyping platform (following the manufacturer's protocol) in a 5-µl volume containing 2.5 µl GeneAmp PCR Master Mix, 0.25 µl 20× MassArray probe and 1 µl genomic DNA with HotStar Taq 500. Allele calling was performed using the Typer4.0 software.

## Results

The descriptive statistics of the PTM-R are presented in Table 1. Table 2 reports the genotype frequencies and information on the number of participants per allelic group, including the minor allele frequencies, number of individuals at each locus and *p*-values for the Hardy-Weinberg equilibrium test. The genotype distributions of all SNPs of *clock*, *AVPR1b* and *OXTR* were in Hardy-Weinberg equilibrium.

**Table 1:** Mean scores and standard deviations of each component of Prosocial Tendencies Measure.

PTM (*n* = 436)	*range*	*Mean*	*SD*
Public	1–5	3.06	0.83
Anonymous	1–5	3.55	0.71
Dire	1–5	4.00	0.70
Emotional	1–5	3.73	0.68
Compliant	1–5	3.77	0.72
Altruism	1–5	3.98	0.68
Total PTM	6–30	22.09	3.24

**Table 2:** *OXTR*, *AVPR1b* and *clock* SNP genotype frequencies.

Gene	SNP	Genotype	mAF	Frequency	*n*	Total	*p*-HWE
*clock*	rs1801260	TT/TC/CC	0.080	0.846/0.149/0.005	369/65/2	436	0.631
	rs6832769	AA/AG/GG	0.279	0.527/0.389/0.084	226/167/36	429	0.515
*AVPR1b*	rs28373064	AA/AG/GG	0.146	0.720/0.269/0.011	313/117/5	435	0.101
*OXTR*	rs1042778	GG/GT/TT	0.075	0.855/0.140/0.005	373/61/2	436	0.769
	rs237887	GG/GA/AA	0.459	0.294/0.494/0.212	128/215/92	435	0.922

Notes: mAF, minor allelic frequency; *p*-HWE, *p-value* of Hardy-Weinberg equilibrium test.

We found a marginal main effect of *OXTR* rs1042778 on Compliant PTM scores [*F* (1, 434) = 3.35, *p* = 0.068, *partial η^2^* = 0.008]; T allele (GT & TT) carriers had lower Compliant PTM scores than those with the GG genotype. No main effects of *clock* rs1801260/rs6832769, *AVPR1b* rs28373064 and *OXTR* rs237887 on the PTM-R or its subscales were detected.

**Figure 1:**
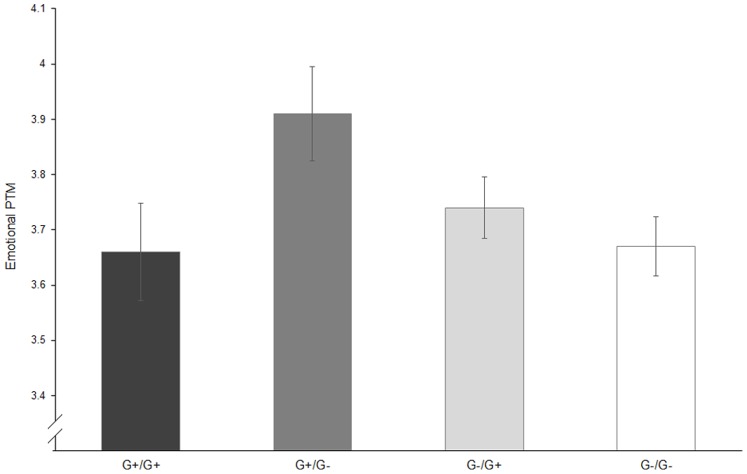
Means and SEMs of Emotional Prosocial Tendencies Measure depending on the interaction of *AVPR1b* rs28373064 and *clock* rs6832769. **G+/G+**: Carriers of the genotype configuration G+/G+, GG or GA for *AVPR1b* rs28373064 and GG or GA for *clock* rs6832769; **G+/G−**: Carriers of GG or GA for *AVPR1b* rs28373064 and AA for *clock* rs6832769; **G−/G+**: Carriers of AA for *AVPR1b* rs28373064 and GG or GA for *clock* rs6832769; **G−/G−**: Carriers of AA for *AVPR1b* rs28373064 and AA for *clock* rs6832769.

Analysis of covariance models revealed a significant interaction effect of *AVPR1b* rs28373064 and *clock* rs6832769 on the Emotional PTM [*F* (1, 425) = 4.88, *p* = 0.028, *partial η^2^* = 0.011], and significance was maintained after Bonferroni correction for multiple testing. Prosociality under emotionally evocative situations for the participants with combined genotype configuration of G+/G− (i.e., GG or GA for *AVPR1b* rs28373064 and AA for *clock* rs6832769, *M_G+/G_*_−_ = 3.91, *SD_G+/G_*_−_ = 0.68, *n* = 62) was the highest compared with the other groups (*M_G+/G+_* = 3.66, *SD_G+/G+_* = 0.77, *n* = 59; *M_G−/G+_* = 3.74, *SD_G−/G+_* = 0.66, *n* = 144; *M_G−/G_*_−_ = 3.67, *SD_G−/G_*_−_ = 0.64, *n* = 164; see Figure 1).

**Figure 2:**
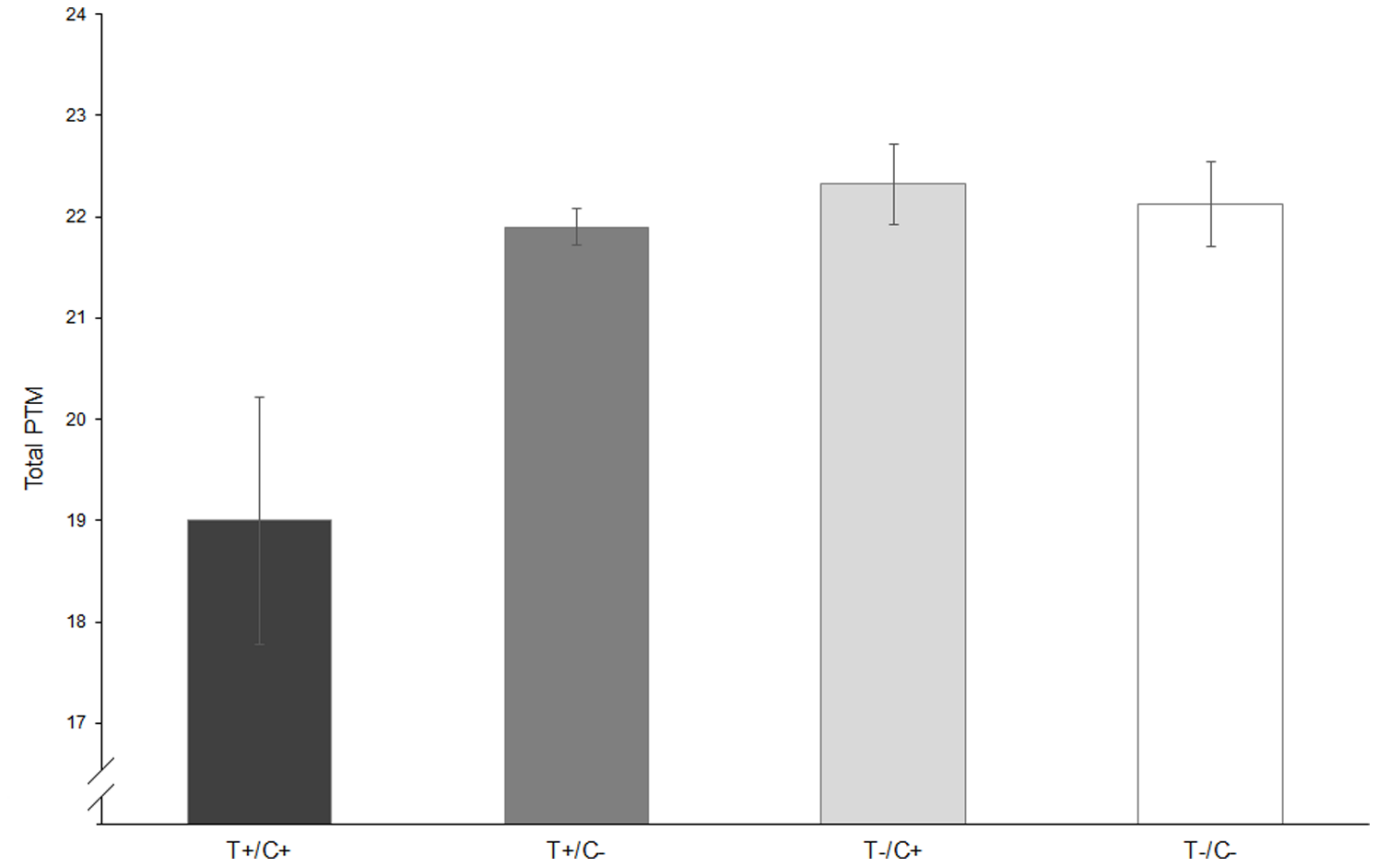
Means and standard deviations of total Prosocial Tendencies Measure depending on the interaction of *OXTR* rs1042778 and *clock* rs1801260. **T+/C+**: Carriers of the genotype configuration T+/C+, GT or TT for *OXTR* rs1042778 and CT or CC for *clock* rs1801260; **T+/C–**: GT or TT for *OXTR* rs1042778 and TT for *clock* rs1801260; **T−/C+**: GG for *OXTR* rs1042778 and CT or CC for *clock* rs1801260; **T−/C−**: GG for *OXTR* rs1042778 and TT for *clock* rs1801260.

There was a statistically significant interaction between *OXTR* rs1042778 and *clock* rs1801260 with regard to total PTM scores [*F* (1,432) = 5.18, *p* = 0.023, *partial η^2^* = 0.012], which was maintained following Bonferroni correction for multiple testing. Carriers of the genotype configuration T+/C+ (i.e., GT or TT for *OXTR* rs1042778 and CT or CC for *clock* rs1801260) showed the lowest total PTM scores (*M_T+/C+_* = 19.00, *SD_T+/C+_* = 3.22, *n* = 7) compared with the other groups (*M_T+/C−_* = 21.90, *SD_T+/C−_* = 3.13, *n* = 56; *M_T−/C+_* = 22.32, *SD_T−/C+_* = 3.08, *n* = 60; *M_T−/C−_* = 22.12, *SD_T−/C−_* = 3.24, *n* = 313; see Figure 2). Further testing revealed that the interaction involving the total PTM scores was influenced mainly by the Anonymous PTM [*F* (1,432) = 5.01, *p* = 0.026, *partial η^2^* = 0.011] and Emotional PTM [*F* (1,432) = 3.94, *p* = 0.048, *partial η^2^* = 0.009].

An additional interaction of *OXTR* rs237887 and *clock* rs6832769 on Public PTM was also detected [*F* (1,424) = 4.63, *p* = 0.032, *partial η^2^* = 0.011], indicating that allele A (AA/AG) of *OXTR* rs237887 was associated with higher Public PTM scores (*Bonferroni p* = 0.049, *partial η^2^* = 0.009) only for those individuals with the *clock* rs6832769 AA genotype. However, this association was not found for the G allele carriers (GG/GA) of *clock* rs6832769 (*Bonferroni p* = 0.288, *partial η^2^* = 0.003; Table 3).

**Table 3:** Means and standard deviations of Public Prosocial Tendencies Measure depending on the interactions of *OXTR* rs237887 and *clock* rs6832769.

	*clock* rs6832769 G+ (GG+GA)		*clock* rs6832769 G− (AA)	
	*n* (%)	*M* (*SD*)	*n* (%)	*M* (*SD*)
*OXTR* rs237887 A+ (AA+AG)	138 (32.2%)	2.99 (0.91)	165 (38.6%)	3.13 (0.77)
*OXTR* rs237887 A− (GG)	65 (15.2%)	3.12 (0.75)	60 (14.0%)	2.90 (0.68)

## Discussion

In the present study, we observed interaction effects of *AVPR1b* rs28373064 and *clock* rs6832769, *OXTR* rs1042778 and *clock* rs1801260, and *OXTR* rs237887 and *clock* rs6832769 on prosociality. These findings suggest that the influences of *AVPR1b* and *OXTR* on prosociality are dependent on the genetic variation of the *clock* gene. Our study also confirms the genotypic effect of *OXTR* rs1042778 on prosociality in complaint situations (i.e., when someone is asked to perform a prosocial behavior), which is in agreement with previous studies, indicating that the GG genotype is associated with high prosociality [15], [25].

Because it is central to circadian rhythms, the *clock* gene determines the biological timing of successful psychological adaption, which partially explains the biological correlates, such as mood and behavior [11]. For example, TT carriers of *clock* gene rs1801260 tend to be morning types and are more conscientious, agreeable and emotionally stable (confirmed as a prosocial personality) compared with C allele carriers, who tend to be evening types, are more impulsive and express emotional apathy [9]. However, we did not find a direct association between the *clock* gene and prosociality, although it has been well established that the formation and maintenance of sleep phase timing and diurnal preferences, such as morning/evening preferences, are regulated by the transcriptional activation of the *clock* gene [7], [11]. We propose that the *clock* gene may affect prosociality [13]. One possible explanation, which is based on the mechanism of CLOCK:BMAL1 heterodimer bindings to E-box enhancers, may be that the *clock* gene influences prosociality through indirect neurophysiological pathways involving other systems, such as OXT-AVP neural pathways, including *OXTR* and *AVPR1b*.

Indeed, our results demonstrated that combinations of *clock* and *OXTR* polymorphisms were associated with prosociality. For example, only the group carrying both risk alleles (CC/CT of *clock* rs1801260 and TT/GT of *OXTR* rs1042778) reported lower levels of prosociality, whereas those subjects with only one risk allele did not demonstrate these lower levels. The C allele of *clock* rs1801260 is characterized by an extreme evening inclination and a high risk of bipolar disorder as well as depression [11], [29], [30]. Additionally, bipolar disorder and depression are closely related to reduced prosocial behavior [31], and the rs1801260 C allele could possibly act as a risk allele of prosociality. Similarly, T allele carriers of *OXTR* rs1042778 display less prosocial behavior than GG carriers [15], suggesting that the T allele of *OXTR* rs1042778 may be another risk allele for prosociality. Our findings show that the combination of both risk alleles at *clock* rs1801260 and *OXTR* rs1042778 lead to lower levels of prosociality, which is consistent with the epistasis effects of genotype on social phenotype. An epistasis effect refers to the phenotypic effect of one locus being dependent on the genotype at a second locus [32], [33]. Previous data have shown that the GG carriers of *OXTR* rs237887 exhibit higher levels of prosociality than the AA carriers [15]. However, the present study found that individuals with the *OXTR* rs237887 A allele (risk allele) more frequently reported prosociality when they were also AA genotype carriers at *clock* rs6832769; the combination of the two genotypes decreased the probability of the reduced prosociality observed in the individuals who carried only the *OXTR* rs237887 risk allele. In general, *OXTR* mRNA expression in the SCN is most likely modulated by *clock* gene, which may affect the efficiencies of *OXTR* and lead to individual phenotypic differences in prosociality.

In addition, we also found that prosociality under emotionally evocative situations is strongest in carriers of the GG or the GA genotype of *AVPR1b* rs28373064 in combination with the AA genotype of *clock* rs6832769. This finding potentially demonstrates epistasis effects on phenotypes [32], Furthermore, the additive effects of genetic variants in both the *clock* and *AVPR1b* genes on prosociality may occur through emotional regulation. Intriguingly, both the *clock* gene and the *AVPR1b* gene are associated with depression and bipolar disorder [18], [34]. Furthermore, the *clock* gene regulates mood-related behaviors via a role in dopamine metabolism [35]; dopamine possibly stimulates AVP release in part based on receptor affinities, interacting to facilitate pair-bond formation [36]. Thus, this combination of *clock* and *AVPR1b* alleles may affect an individual’s emotional activation levels synchronously, leading to variations in prosocial behavior. Moreover, previous studies have reported many combined effects of circadian clock genes (or components) and other genes (or components). For example, clock genes influence mood by regulating monoamine oxidase A in dopamine metabolism [35] and resistance to weight loss in combination with the *sirt1* gene [37]. It appears that the *clock* gene influences the expressions of other genes through the processes of cell metabolism and extracellular signaling [38], which may represent the underlying mechanism of the effects of the *clock* gene on social behavior.

In summary, we have provided the first evidence that human prosociality is affected by the combined effects of genetic variations in the *clock* gene and genes involved in the OXT and AVP systems. A limitation of our study was the small size of the combined group possessing the C allele of *clock* rs1801260 and the T allele of *OXTR* rs1042778 (*n* = 7, 1.6%); it is possible that the C allele frequency of *clock* rs1801260 is minor, especially within the Asian population (i.e., 8.0% in Han Chinese; 8.3–9.1% in Koreans) [39], [11]. The focus on male participants allowed us to avoid potential gender bias in our results [40], but it also limited the generalizability of the present findings. Further replication of these findings in independent study samples (including female subjects) and a meta-analysis to confirm the combined gene effects are needed. Future studies using situational prosocial behavior tasks will be necessary to confirm our results, which were based on self-reported data. The mechanisms underlying the observed associations of these combinations remain to be elucidated. The *clock* gene may be a point through which changes in cellular energy metabolism influence the functioning of the OXT and AVP systems. Future studies are necessary to determine these mechanisms.

In conclusion, this study demonstrates a link between *clock* genotypes and prosociality phenotypes, indicating that the combined effects of genetic variations in the *clock* gene and genes involved in the OXT and AVP systems may contribute to human prosociality. In addition, we provide genetic evidence that further explains the mechanisms underlying prosocial behavior.

## Supporting Information

Data S1 **Data S1 is the raw data of our paper, including the raw data of each dimension of PTM scale, the genotypes of each participant as well as the demographic variables.**
(SAV)
